# dATP/ATP, a Multifunctional Nucleotide, Stimulates Bacterial Cell Lysis, Extracellular DNA Release and Biofilm Development

**DOI:** 10.1371/journal.pone.0013355

**Published:** 2010-10-14

**Authors:** Chuanwu Xi, Jianfeng Wu

**Affiliations:** Department of Environmental Health Sciences, University of Michigan, Ann Arbor, Michigan, United States of America; University of Medicine & Dentistry of New Jersey-New Jersey Medical School, United States of America

## Abstract

**Background:**

Signaling by extracellular adenosine 5′-triphosphase (eATP) is very common for cell-to-cell communication in many basic patho-physiological development processes. Rapid release of ATP into the extracellular environment from distressed or injured eukaryotic cells due to pathogens or other etiological factors can serve as a “danger signal”, activating host innate immunity. However, little is known about how or whether pathogenic bacteria respond to this “danger signal”.

**Methods and Principal Findings:**

Here we report that extracellular dATP/ATP can stimulate bacterial adhesion and biofilm formation via increased cell lysis and extracellular DNA (eDNA) release. We demonstrate that extracellular dATP/ATP also stimulates bacterial adherence *in vitro* to human bronchial epithelial cells.

**Conclusions and Significance:**

These data suggest that bacteria may sense extracellular dATP/ATP as a signal of “danger” and form biofilms to protect them from host innate immunity. This study reveals a very important and unrecognized phenomenon that both bacteria and host cells could respond to a common important signal molecule in a race to adapt to the presence of one another. We propose that extracellular dATP/ATP functions as an “inter-domain” warning signal that serves to induce protective measures in both Bacterial and Eukaryotic cells.

## Introduction

Signaling by extracellular adenosine 5′-triphosphase (eATP) plays an important role in diverse patho-physiological processes and is used ubiquitously amongst eukaryotes as an intercellular signal [1]. Recent work demonstrated that eATP acts as a signal molecule to activate host inflammatory and immune response [2], [3], [4]. Pathogens and other etiological factors can cause distress or injury of host cells and stressed or injured cells release ATP via lytic or non-lytic pathways. A rapid increase of ATP concentration in the extracellular space acts as an early and sensitive sign of cellular stress (“danger signal”) and alerts the immune system of an impending danger due to exogenous and endogenous causes [5], [6] to activate the quick inflammatory response to clear invading pathogens. However, little is known about whether and/or how bacterial pathogens can sense and respond to the same “danger signal” (eATP) to avoid being removed by the host innate immune response.

Biofilm formation is one of the mechanisms bacteria use to survive in adverse environments [7], [8], [9], [10]. Biofilm is an accumulation of bacterial cells encapsulated in an extracellular polymer matrix that adheres to surfaces and formation of biofilms can be induced in response to stress conditions including sublethal concentrations of aminoglycoside antibiotics [11], osmotic stress [12], high metal concentration, extreme pH and temperature, the addition of xenobiotics, antibiotics, and oxygen [13]. The matrix is composed of extracellular polymeric substances (EPSs) which include polysaccharides, proteins, nucleic acids and amphiphilic polymers [14], [15], which are secreted by microorganisms into their environment [16]. The EPS is involved in the establishment of stable arrangements of microorganisms in biofilms [17] and protect embedded bacteria from attach of antimicrobials and human immune system [7], [8], [9]. However, little is known about what factors in eukaryotic host that affect bacterial biofilm formation or dispersal. Here we report that extracellular dATP/ATP can stimulate bacterial adhesion and biofilm formation via increased cell lysis and extracellular DNA (eDNA) release, which could be one of the mechanisms that bacteria employ to protect them from host innate immunity. We hypothesize that extracellular dATP/ATP functions as an “inter-domain” warning signal that serves to induce protective measures in both Bacterial and Eukaryotic cells.

## Results

### dATP/ATP stimulates bacterial biofilm formation

**Figure 1:**
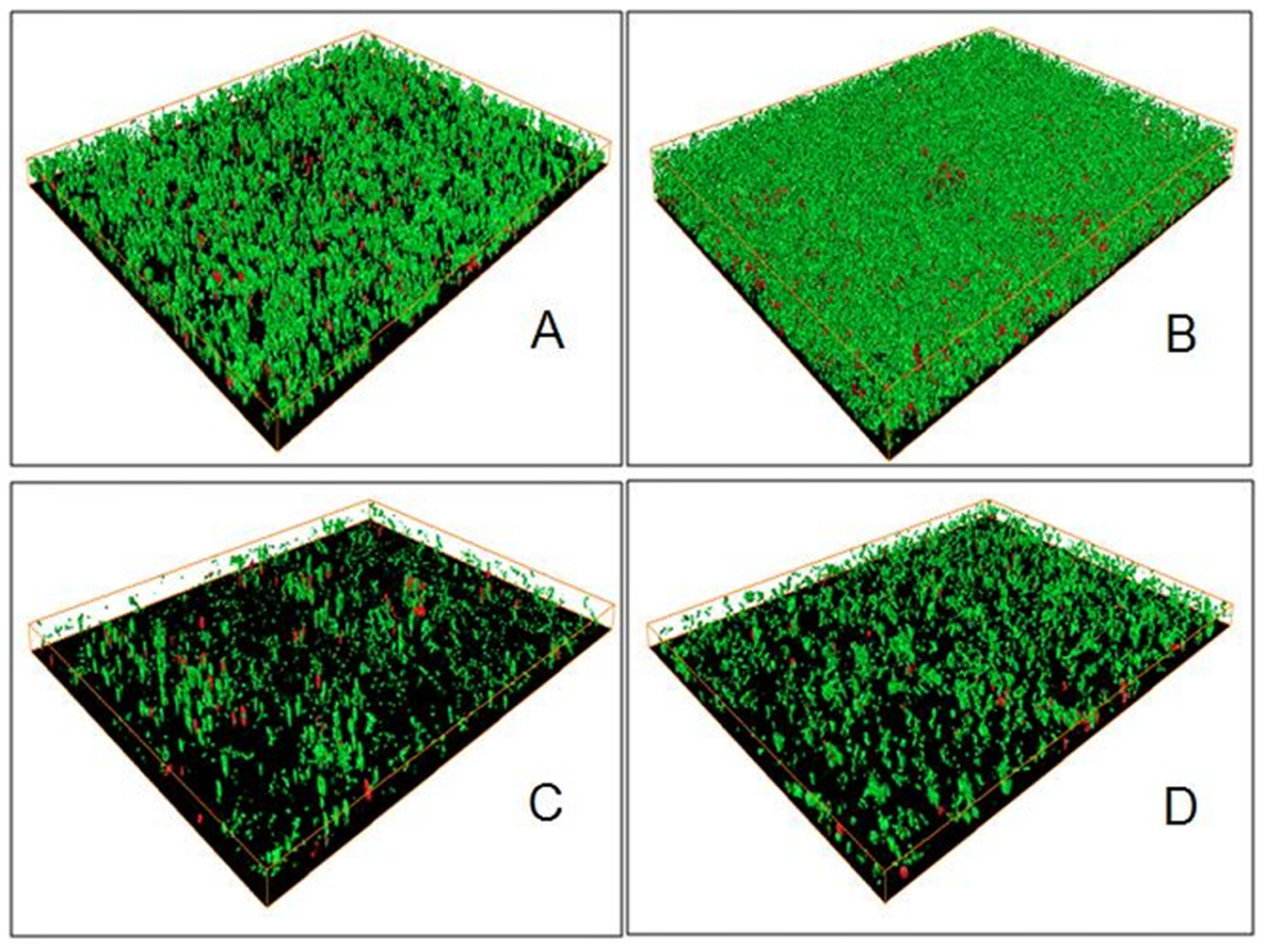
Confocal laser scanning micrographs of biofilms developed by *E. coli* K-12 in 10% LB broth with different reagents. A, control, without any additional reagents; B, with 400 µM of dATP;, C, with 40 U/ml of DNase I; and D, with 200 mU/ml of Apyrase. Biofilms were examined after 16 hrs incubation after inoculation at 30°C in a 96-well microtiter plate. Bacterial cells were stained with Bacterial LIVE/DEAD staining dyes and viable cells shown green and dead or membrane damaged cells shown red in the images.

**Figure 2:**
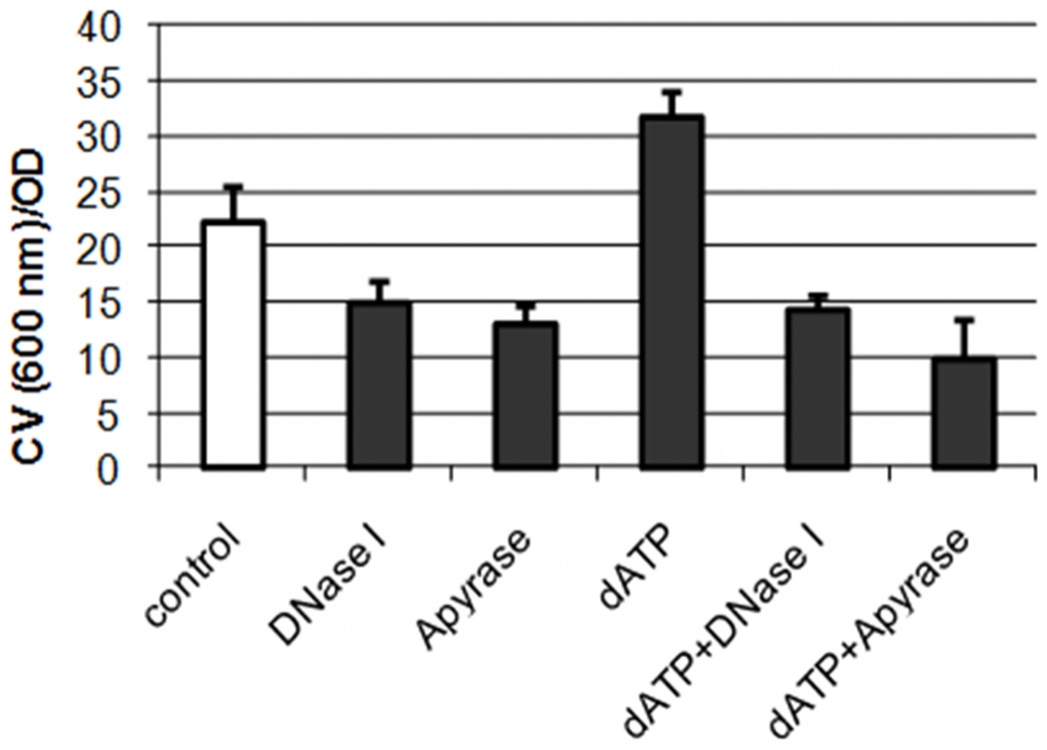
Quantification of biofilms developed by *E. coli* K-12 in 10% LB supplemented with different reagents: no addition of any reagents (control); DNase I (40 U/ml); Apyrase (200 mU/ml); dATP (400 µM); dATP (400 µM) and DNase I (40 U/ml); or dATP (400 µM) and Apyrase (200 mU/ml). Biofilms were examined after 16 hrs incubation after inoculation at 30°C in a 96-well microtiter plate. Crystal violet staining assay was used to determine biofilm biomass.

**Figure 3:**
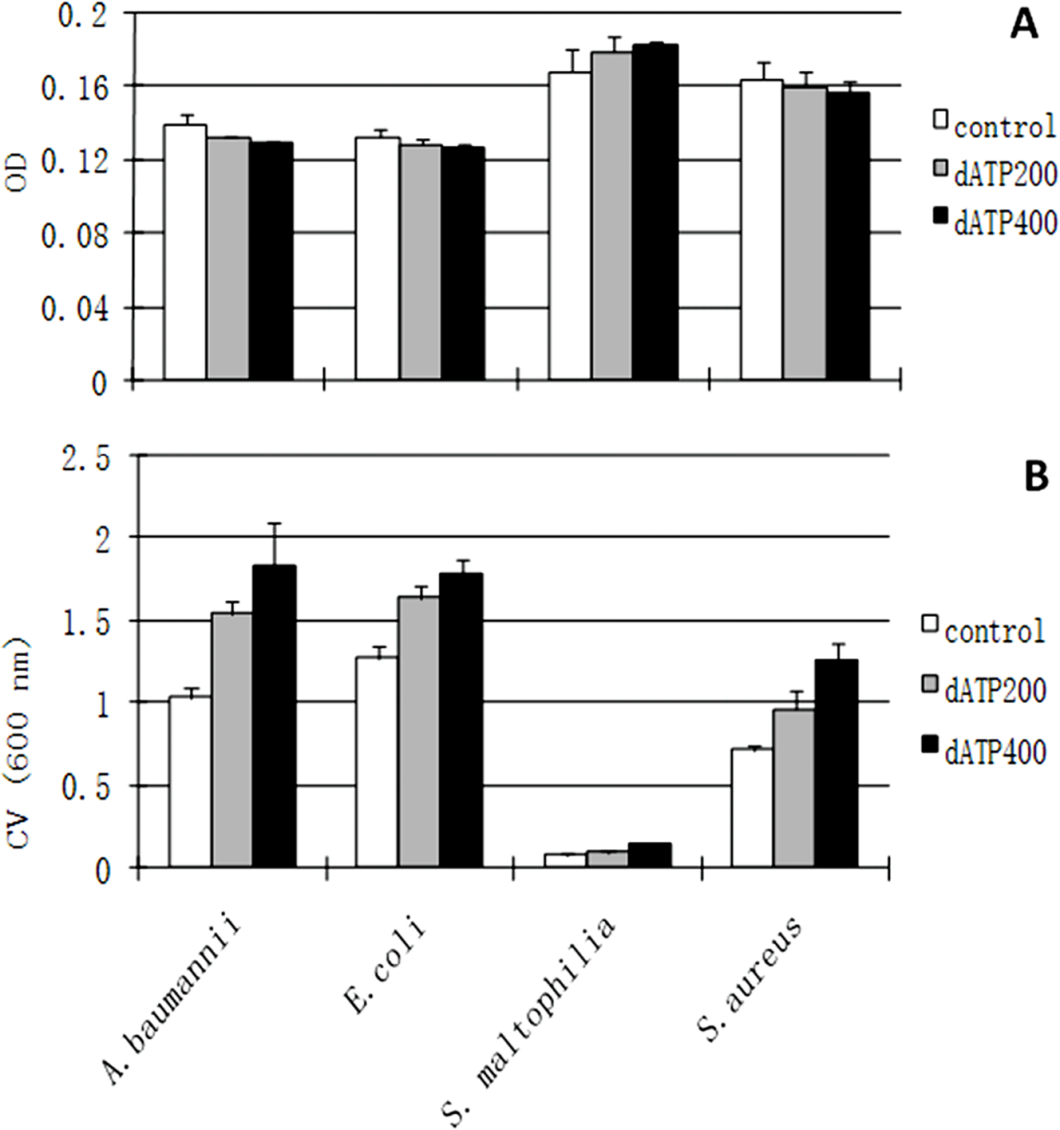
Growth and biofilm formation of different strains in the absence (control) or the presence of dATP (dATP200, 200 µM of dATP; dATP400, 400 µM of dATP). 1% overnight culture were inoculated in 100 µl of 10% LB broth in wells of 96-well microtiter plate with different concentration of dATP, 10% LB without dATP was set as control. A, growth OD was measured by reading absorbance at 600 nmafter 16 hours incubation at 30°C. B, biofilm biomass was recorded by reading absorbance at 600 nm after crystal violet staining. Statistical analysis of three independent experiments showed significant increase of biofilm formation by all strains in the presence of dATP.

We first tested the effect of dATP/ATP on *Escherichia coli* biofilm formation and found that the addition of dATP or ATP (400 µM) in culture media, which is in the range of physiological eATP concentration, can stimulate *E. coli* initial attachment and biofilm formation on the glass surface (Figures 1 and 2). Dense, thick and carpet-like biofilm was formed by *E. coli* cells in the presence of dATP (Figures 1. B) compared to loosen carpet-like biofilm without dATP treatment (Figures 1. A). Attached biofilm biomass increased by about 50% (Figure 2) with dATP treatment. dATP treatment did not have observable effect on *E. coli* growth in suspended cultures (Figure 3. A). Treatment with apyrase (200 mU/ml), an enzyme that catalyses the hydrolysis of ATP to yield AMP and inorganic phosphate [18], partially arrested *E. coli* biofilm development (Figures 1. D). Only dispersed microcolonies were observed on the surface and there was about 40% reduction of biofilm biomass compared to without the apyrase treatment (Figure 2), suggesting that eATP is required for biofilm formation. In addition, *E. coli* developed about 50% less biofilm when treated with dATP (400 µM) and apyrase (200 mU/ml) together (Figure 2), demonstrating it is dATP that stimulates *E. coli* biofilm development. We also tested the effects of other three nucleotides (dCTP, dGTP and dTTP) on *E. coli* biofilm formation and found that dGTP has a similar effect with less extent and no effects from dCTP and dTTP treatment was found (data not shown). dATP can also stimulate biofilm formation of several other pathogenic bacterial strains including *Acinetobacter baumannii*, *Stenotrophomonas maltophilia* and *Staphylococcus aureus* (Figure 3. B), without observed impact on their growth (Figure 3. A), although these strains have different biofilm formation capacity. This data suggests that the stimulus effect of dATP on bacterial biofilm formation might be common among different bacterial strains.

### Extracellular DNA is involved in the stimulus effect of dATP/ATP

**Figure 4:**
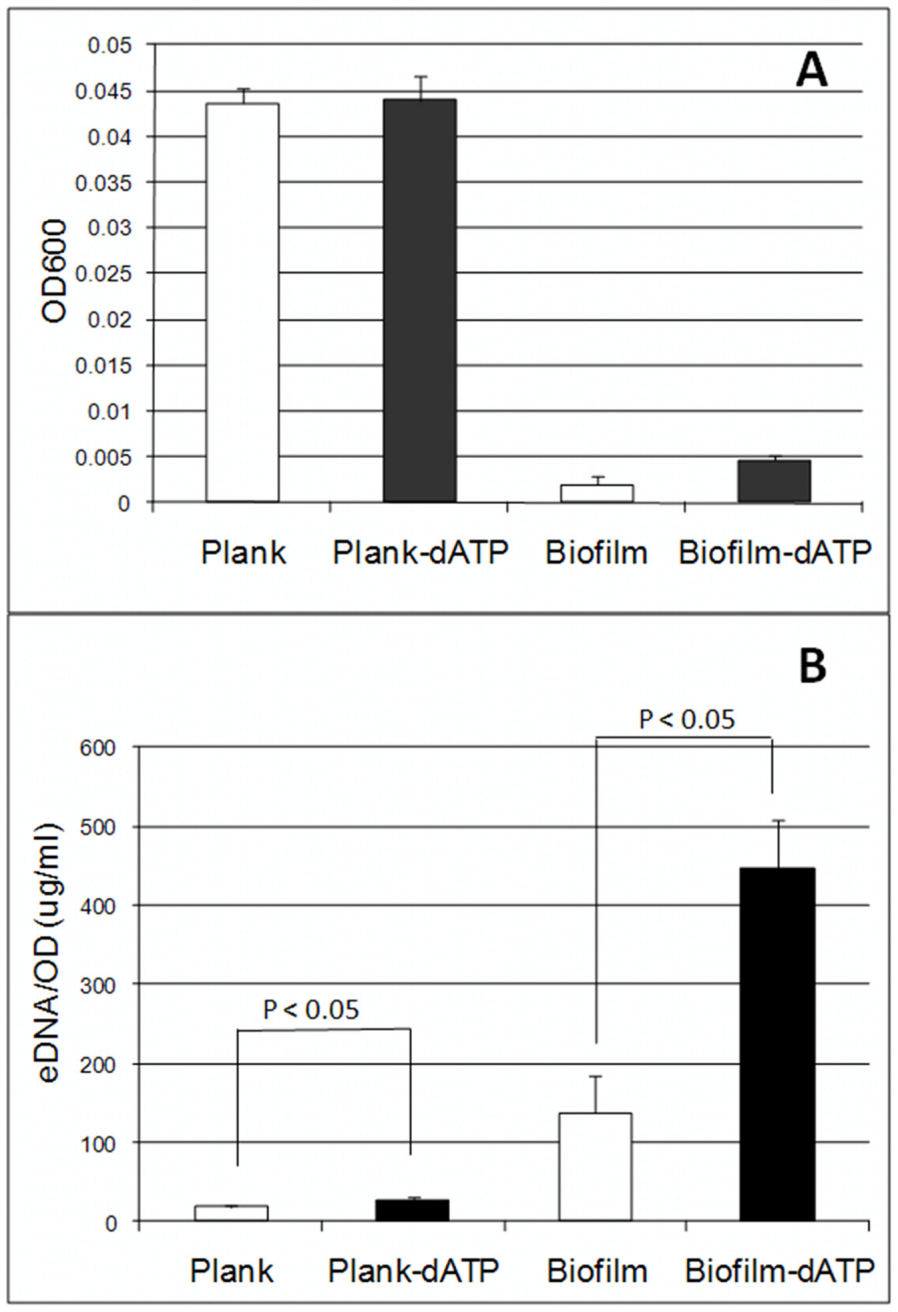
Growth of *E.coli* K-12 in suspensions and biofilms and eDNA released in suspended cultures and biofilm matrix in 10% LB supplemented with or without dATP (400 µM). A, growth of *E.coli* K-12 in suspensions and biofilms; B, eDNA in cultures and biofilm matrix. eDNA was extracted using the method we developed (see the Materials and Methods section) and quantified using the PicoGreen dsDNA Quantitation Kit (Molecular Probes, Invitrogen). P values from statistical analysis of three independent experiments were shown in the graph. Student T-test was used for statistic analysis.

**Figure 5:**
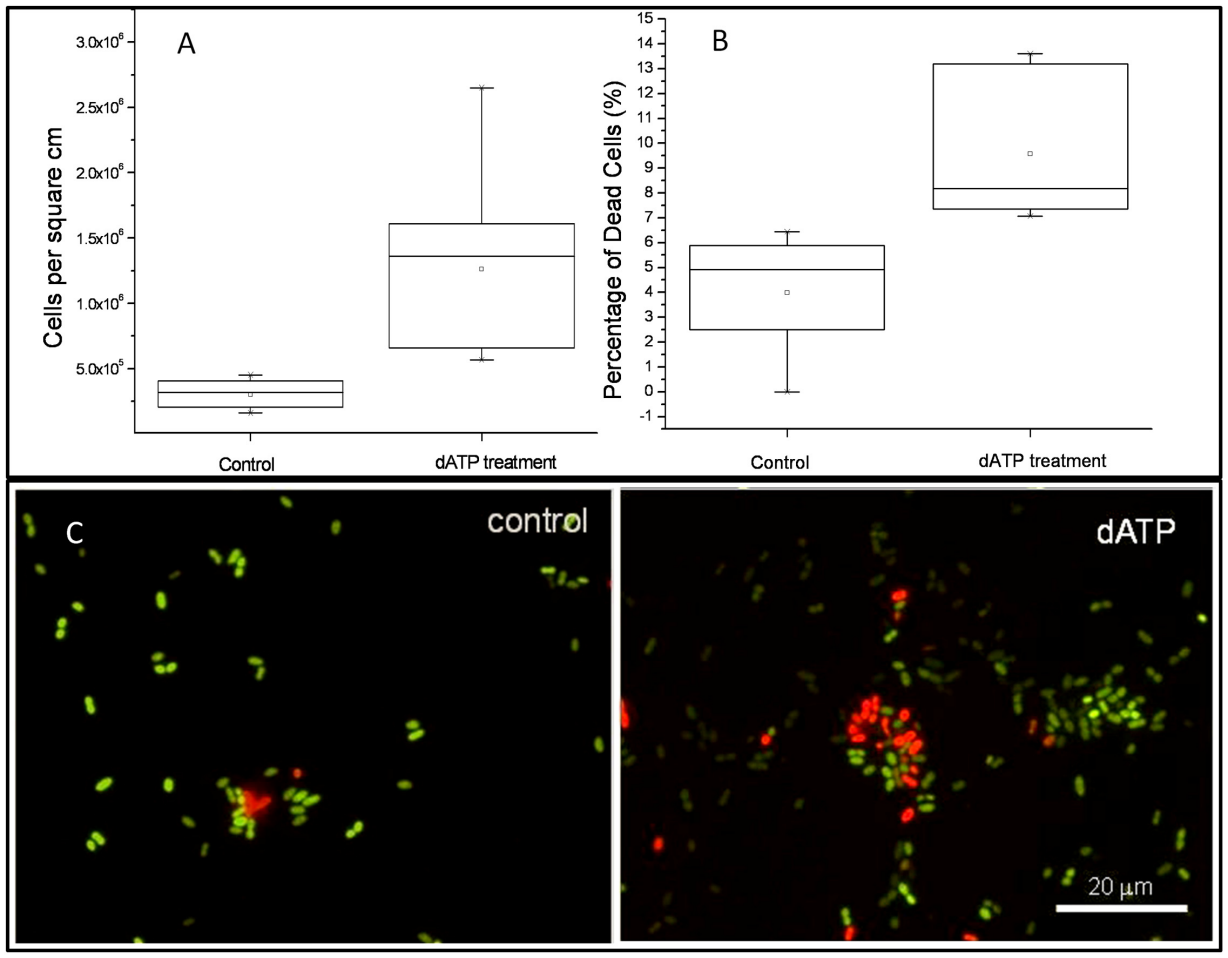
Quantification and fluorescent micrographs of cell attachment and cell lysis on glass surfaces of the *E. coli* strain. *E. coli* (1–1.5×10^6^ CFU/mL) was incubated in a petri dish with glass coverslips submerged in 10% LB supplemented with or without dATP (400 µM) at 30°C with gentle shaking for 2 hours. Coverslips were stained with Bacterial Live/Dead staining dyes before being observed under an epi-fluorescent microscope. A, number of bacterial cells attached to the glass surfaces; B, percentage of dead or membrane damaged cells; C, Representative micrographs shown attached live cells (green) and dead cells (red).

We further studied how bacteria cells respond to eATP with a focus on the link with extracellular DNA (eDNA). eDNA is one of the major components of EPS [19], [20] that bacteria release as part of the biofilm matrix and is critical for biofilm development, e.g., facilitates initial bacterial attachment to abiotic or biotic surfaces and maintain the structure of mature biofilms [20], [21], [22], [23], [24], [25], [26], [27]. We first tested the role of eDNA in *E. coli* biofilm development. DNase treatment arrested *E. coli* biofilm formation (Figure 2), and only dispersed microcolonies attached on the glass surface can be observed (Figure 1. C). Attached *E. coli* biofilm biomass was reduced about 50% with DNase treatment (Figure 2). *E. coli* biofilm formation was completely arrested by the treatment with dATP together with DNase (Figure 2), suggesting that dATP-mediated stimulation of biofilm formation involves eDNA. Thus we tested whether dATP treatment could induce eDNA release and found that dATP treatment increased about 48% of eDNA release in *E. coli* planktonic cells or about 200% of eDNA release in the biofilm matrix (Figure 4. B), without impacting *E. coli* growth in suspended cultures (Figure 4. A). Moreover, the percentage of membrane-damaged cells attached on the glass surface after incubation for two hours increased more than 2-fold in the presence of dATP treatment (9.58% with dATP treatment vs. 3.99% without dATP treatment; p value <0.01) and total attached cells increased from 3.02×10^5^ cells/cm^2^ to 7.97×10^5^ cells/cm^2^ (Figure 5). These data suggest that exogenous dATP can induce cell lysis and eDNA release, leading to increased bacterial attachment and biofilm formation on abiotic surfaces.

### dATP stimulates bacterial adherence to human epithelial cells

**Figure 6:**
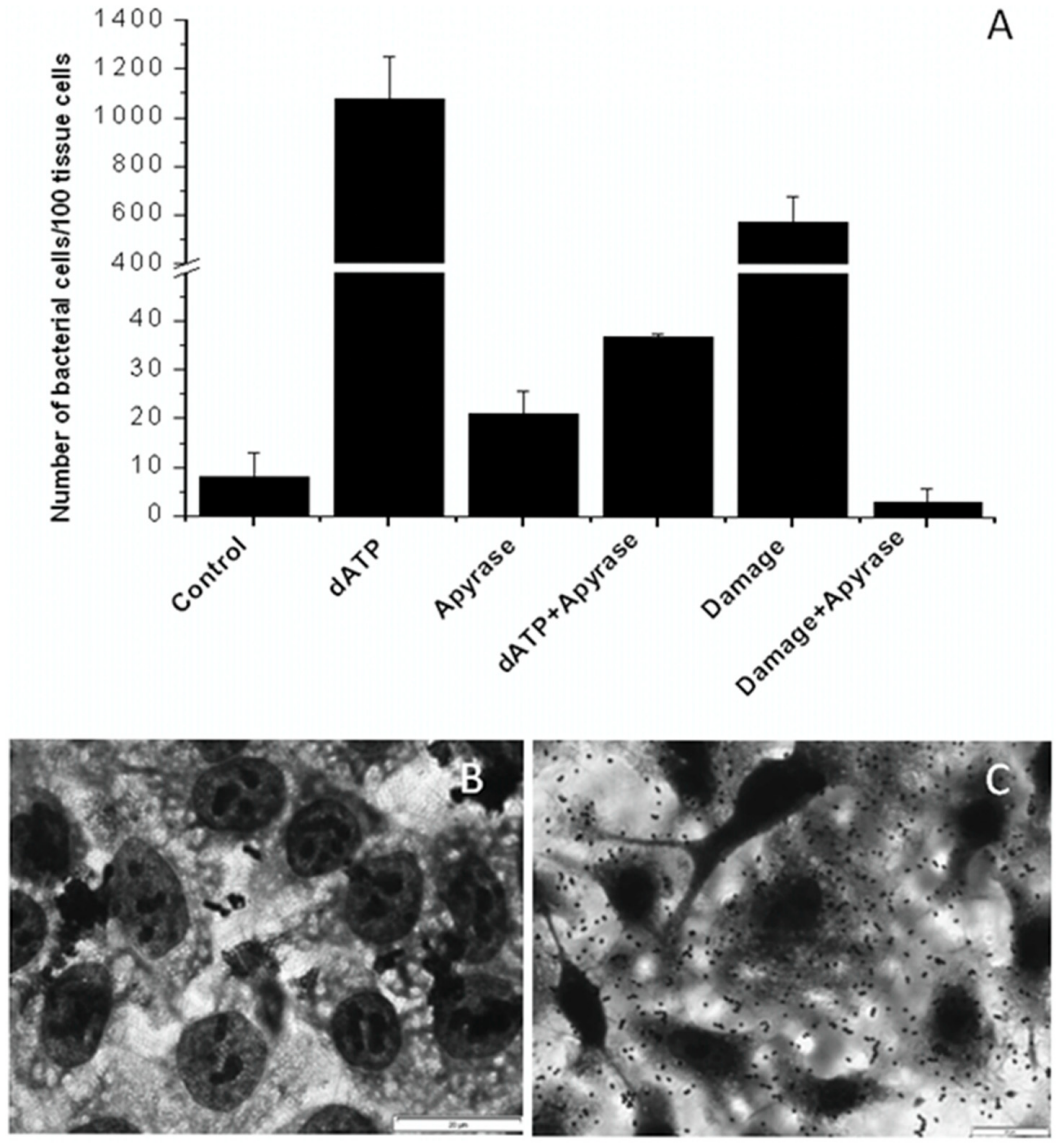
Quantification and light micrographs demonstrating the simulative effect of dATP on adherence of *A. baumannii* to human bronchial epithelial NCI-H292 cells. A, Number of *A. baumannii* cells adhered to the monolayer of 100 human bronchial epithelial NCI-H292 cells after 1 hour incubation at 37°C with 5% (v/v) CO_2_ in the RPMI 1640 medium with different treatments: Control (no treatment); dATP (medium supplemented 400 µM of dATP; Apyrase (medium supplemented with 200 mU/ml of Apyrase; Damage (around 5% of a monolayer of the epithelial cells was damaged); and a combination of two treatments. Three independent experiments were performed for each treatment and standard deviations of three treatments were included. B, Representative light micrograph of *A. baumannii* cells adhered to the epithelial cells after incubation in the RPMI 1640 medium without supplementing dATP;and, C, with supplementing dATP. (Objective 60×).

Interestingly, in early studies of animal models of *Pseudomonas* infection, few bacteria were found to be adherent unless the respiratory epithelium was injured by acid or viral infection [28]. Wound or cell damage in epithelial cells can induce rapid release of ATP [29]; therefore, we further tested whether eATP is involved in bacterial adherence to epithelial cells. We used an *in vitro* cell culture model of human bronchial epithelial NCI-H292 cells and *A. baumannii* was chosen for this test. *A. baumannii* is an emerging bacterial pathogen and is responsible for the majority of nosocomial pulmonary infections [30], [31]. We found that supplementing the media with 400 µM of dATP increased *A. baumannii* adherence about 100-fold one hour after incubation (1080 bacterial cells/100 tissue cells with dATP treatment vs. 8 bacterial cells/100 tissue cells without dATP treatment), by counting adhered bacterial cells on the monolayer of the bronchial epithelial cells, and promoted bacterial aggregate formation on human bronchial epithelial cells (Figure 6). The data was confirmed by counting viable *A. baumannii* cells adhered to the monolayer of human bronchial epithelial cells in the petri dish (8.79×10^5^ CFU/cm^2^ (±6.63×10^4^ CFU/cm^2^) vs. 1.6×10^4^ CFU/cm^2^ (±1.2×10^3^ CFU/cm^2^) without dATP treatment). The similar level of increased bacterial adherence was observed when a small portion of human cells were physically damaged (Figure 6). Rapid increase of ATP into the media was likely occurred due to this damage. Increased bacterial adherence either due to exogenous added dATP or cell damage was completely arrested by the apyrase (200 mU/ml) treatment (Figure 6). These data clearly demonstrated that bacteria respond very quickly to exogenous dATP or ATP released from damaged cells and increase adherence to human epithelial cells.

## Discussion

Bacteria and their eukaryotic hosts have coexisted for millions of years and bacteria could be beneficial, neutral or detrimental to their hosts. Eukaryotic hosts and bacteria developed different mechanisms to maintain a fine balance and communicate with each other through different signals, e.g. hormonal signals [32]. The concept of using eATP as an extracellular messenger was first proposed over 30 years ago [1] and it was recently found it can act as a “danger signal” to alert and activate host immune system to prevent bacterial infections [2], [3], [4]. In this study, we tested whether bacteria could take advantage of this signal molecule released either from the host and/or themselves for their benefits when interact with their eukaryotic host.

We first tested the response of bacteria to exogenous added dATP/ATP. It was found that dATP can stimulate biofilm formation by bacterial strains tested (Figures 1, 2 and 3). Four bacterial species including Gram-negative bacteria *E. coli*, *A. baumannii*, *S. maltophilia* and Gram-positive bacterium *S. aureus* have been tested with more detailed studies on *E. coli* and *A. baumannii*. *E. coli* is a well studied model organism and available knowledge and database about gene regulations in this bacterium will be useful for further studies on extracellular ATP signaling. *A. baumannii* is a notorious nosocomial pathogen that causes a range of infections, including respiratory and urinary tract infections, meningitis, endocarditis, wound infections and bacteraemia, especially in intensive care unit patients [33], [34]. It becomes a serious concern of hospital acquired infection because of the emergence of multidrug-resistant strains of this specie and some of them are pan-resistant to antimicrobial agents. These four bacterial species respond similarly to exogenous dATP/ATP treatment on biofilm formation led us to propose that the stimulus effect of dATP/ATP on biofilm formation could be common among other bacterial species; furthermore, more tests on much broader representative species would strengthen this hypothesis.

Exogenously added dATP increased biofilm formation and this effect was removed by the apyrase treatment (Figures 1 and 2). Apyrase is an enzyme that catalyses the hydrolysis of ATP to yield AMP and inorganic phosphate [18]. Apyrase treatment not only eliminated the effect of exogenously added dATP but also further arrested biofilm formation to a similar level of DNase treatment. It has been demonstrated that extracellular DNA is a major component of biofilm matrix and required for biofilm formation by different bacterial strains, which was initially tested in a study which found that DNase treatment arrested biofilm formation [21]. The similar effect caused by the apyrase treatment as the DNase treatment suggest that extracellular ATP might be also required for biofilm formation, although it is not clear about mechanisms of the role of extracellular ATP in biofilm formation.

Further tests in this study found a link between the effect of dATP/ATP treatment and extracellular DNA on biofilm formation. It was found that dATP/ATP treatment induced cell lysis and eDNA release both in suspended cultures and biofilms (Figure 4), which suggest that extracellular DNA probably is involved in the stimulus effect of dATP/ATP on biofilm formation. It is not clear, though, about how bacterial cells sense extracellular dATP/ATP and how dATP/ATP causes cell lysis and DNA release. In eukaryotic cells, there are two type of purinoreceptors (P2X and P2Y) which can be activated by ATP [35]. Although we haven't found homologous receptors in *E. coli* using the BLAST search(data not shown), it is not likely due to toxic effects of ATP on bacterial cells for cell lysis and DNA release since exogenously added ATP doesn't affect *E. coli* growth. Thus we believe that *E. coli* cells may sense extracellular ATP and initiate certain cellular processes for cell lysis and eDNA release, a process might be similar to eukaryotic programmed cell death. Other cellular processes in addition to cell lysis and eDNA release may also be involved in the stimulus effect. Further studies are warranted to elucidate molecular mechanisms of this effect of ATP on biofilm formation.

Recent study found that extracellular dATP/ATP can act a “danger signal” to alert and activate host immune system to prevent bacterial infection, which led us to use an *in vitro* cell culture test to study whether extracellular dATP/ATP could promote bacterial adherence to eukaryotic cells. We found that exogenously added dATP or ATP from damaged cell increased bacteria adherence and this effect was attenuated by apyrase treatment (Figure 6). The data suggest bacterial cells can adhere to eukaryotic cells in response to extracellular dATP/ATP. In this experiment, it was also found that dATP treatment changed the morphology of human bronchial epithelial cells (Figure 6. C), suggesting that host cells also respond to exogenously added dATP. This change may also contribute to increased bacterial adherence but clearly could not the sole factor. This study and previous studies in literatures led us to propose that dATP/ATP may act as an “inter-domain” signal molecule that regulates the interaction between Eukaryotic and Bacterial cells. Further *in vivo* animal model studies, e.g. lung or wound infection model, to test ATP treatment and/or apyrase treatment on the induction of proinfammatory cytokins in host and bacterial colonization and infection would provide more in depth understanding of this “inter-domain” signaling.

In conclusion, we found bacteria may sense extracellular dATP/ATP as a “danger signal” and initiate cell lysis and eDNA release resulting in increased bacterial adherence and biofilm formation for the purpose of protecting them from the activated host innate immunity. This study reveals a potentially very important and unrecognized phenomenon that both bacteria and host cells can respond to a same important signal molecule in a race to adapt to the presence of one another. Further study of this phenomenon will lead to more comprehensive understanding of bacteria-host interactions and bacterial pathogenesis and will likely lead to development of a novel approach to prevent bacterial infections and to treat related human diseases.

## Materials and Methods

### Bacterial strains, media and enzymes

*E.coli* K-12 BW25113, *Acinetobacter baumannii* ATCC 17978, *Stenotrophomonas maltophilia*, and *Staphylococcus aureus* ATCC 25923 were grown on rich medium (Luria-Bertani; LB) overnight at 37°C with shaking at 250 rpm. Biofilm were grown at 30°C in 10% of LB medium in the wells of 96-well microtitre plates. DNase I and Apyrase were obtained from New England Biolabs. dATP and ATP were obtained from Promega (Promega Company, Madison, WI). All other chemicals, unless otherwise specified, were reagent grade or higher (Sigma-Aldrich. St. Louis, MO).

### Biofilm growth and harvesting

Development of biofilms was performed in 96-well microtiter plates following the method as described previously [36]. Quantification of biofilm biomass was obtained by using the method according to O'Toole *et al.* [37]. 1% of overnight culture was inoculated into the wells of 96-well microplate containing 100 µl of 10% LB media supplemented with different concentration of dATP/ATP, Apyrase or DNase accordingly. After 16 h of incubation at 30°C, 15 µl of a 1% solution of crystal violet (CV) was added to each well to stain the cells for 15 min. Then, the wells were rinsed thoroughly with PBS buffer three times. Biofilm formation was quantified by the addition of 100 µl of 95% ethanol to each CV-stained microplate well, and the absorbance was determined with a plate reader at 600 nm (Synergy HT, Bio-Tek. Winooski, VT).

### Image acquisition and analysis

All microscopic observations and image acquisitions were performed with Olympus IX71 equipped with detectors and filter sets for monitoring of SYTO-9 and propidium iodide (PI). Images were obtained using a 60× objective. Biofilm samples were stained with LIVE/DEAD BacLight Bacterial Viability Kits (cat. L7007, Invitrogen) following the instructions in the manual. Three dimensional images were performed with an Olympus Fluoview™ FV1000 confocal microscope (Olympus, Markham, Ontario) with Melles Griot Laser supply and detectors and filter sets for monitoring SYTO 9 and PI fluorescence. Images were obtained using a 60× objective. Simulated 3D images were reconstructed using the Amira software package (Amira, San Diego, CA).

### Extraction and quantification of extracellular DNA

Biofilms were developed as mentioned above for 16 hours and biofilm cells were resuspended with 0.9% NaCl solution, homogenized for 1 min and were ready for eDNA extraction. Planktonic cells were incubated at 30°C for 8 hours with a 1% of inoculation and cells were washed with fresh 10% of LB broth three times before resuspended in the same volume of 10% of LB broth or 10% LB broth supplemented with 400 µM of dATP. The cells solutions were then incubated at 30°C for another 2 hour and were ready for eDNA extraction. Biofilm cells suspension and planktonic cultures were treated as previously reported [38] for DNA extraction. Briefly, cell solution was treated with Dispersin B (20 µg/ml) at 37°C for 30 min followed by treatment of proteinase K (5 µg/ml) for another 30 min at 37°C. Treated cell solution was filtered through a 0.2 µm syringe filter (PES, Water & Process Technologies), elute was used to extract the extracellular DNA by using ethanol precipitation. DNA was dissolved in 50 µl of sterile MilliQ water and the concentration of DNA was measured by using the PicoGreen dsDNA Quantitation Kit (Molecular Probes, Invitrogen).

### Cell Attachment Assay

1% of overnight *E. coli* culture was inoculated in 20 ml of 10% LB broth in petri dish with glass coverslips in absent or present of dATP. Petri dish plate was incubated at 30°C with shaking at 80 rpm. After 2 hours, coverslips were taken out, gently washed with PBS buffer three times and stained with LIVE/DEAD BacLight Bacterial Viability Kits for cell number counting. Three coverslips were observed for each test and five randomly chosen images were taken from each coverslip. Number of attached cells was counted from each image and percentage of membrane damaged cells (red cells) was calculated. Student T-test was used for statistic analysis.

### *In vitro* bacterial adherence assay

Bacterial adherence assay was performed following a method described previously [39]. Human bronchial epithelial cell line NCI-H292 (ATCC CRL-1848; American Tissue Culture Collection, Rockville, MD, USA) were cultured in a petri dish at 37°C in RPMI 1640 medium (Gibco BRL, Grand Island, NY, USA), supplemented with 25 mM HEPES, 2 mM L-glutamine, penicillin G 100,000 U/L, streptomycin 50 mg/L and 10% (v/v) of fetal bovine serum (Gibco BRL), in a humidified atmosphere containing 5% (v/v) of CO_2_.

Two ml of fresh NCI-H292 (1×10^5^ cells/ml) cell solution were transferred to each well of a 12-well plate, which contained a 13-mm-diameter plastic coverslip (Thermanox, Nunc, Rochester, NY, USA) in each well. The cells were incubated at 37°C for about 3 days until cells covered about 90% of coverslip and were then washed with phosphate buffered saline (PBS) three times. Overnight culture of *Acinetobacter baumannii* ATCC 17978 were washed and mixed with reagents (400 µM of dATP, or 200 mU/ml of apyrase) in PBS if necessary. Each cell monolayer was infected with 1 mL of bacterial suspension (1×10^8^ CFU/mL) and incubated for 60 min at 37°C in a CO_2_ 5% v/v atmosphere. For damaged cell assays, NCI-H292 cells (around 5%) in the central area of plastic coverslips were damaged by tip of a knife before the infection performed. After infected by *Acinetobacter baumannii* ATCC 17978, plastic coverslips were washed with PBS buffer three times to remove non-adherent bacteria. Some of coverslips were mixed with 3 ml of PBS buffer and homogenized for 30 sec. Mixtures were diluted 10-fold serially for plate counting. Some coverslips were fixed in 100% of methanol for 20 min before being stained in a Giemsa staining solution for 30 min at room temperature. The coverslips were air-dried, mounted and observed under a light microscope with a ×60 objective len. The number of bacteria adhering to 100 cells was determined. Three independent experiments were performed for each treatment.
